# Sex-Biased Gene Expression on the Avian Z Chromosome: Highly Expressed Genes Show Higher Male-Biased Expression

**DOI:** 10.1371/journal.pone.0046854

**Published:** 2012-10-03

**Authors:** Sara Naurin, Dennis Hasselquist, Staffan Bensch, Bengt Hansson

**Affiliations:** Department of Biology, Lund University, Lund, Sweden; University of Maryland School of Medicine, United States of America

## Abstract

Dosage compensation, the process whereby expression of sex-linked genes remains similar between sexes (despite heterogamety) and balanced with autosomal expression, was long believed to be essential. However, recent research has shown that several lineages, including birds, butterflies, monotremes and sticklebacks, lack chromosome-wide dosage compensation mechanisms and do not completely balance the expression of sex-linked and autosomal genes. To obtain further understanding of avian sex-biased gene expression, we studied Z-linked gene expression in the brain of two songbirds of different genera (zebra finch, *Taeniopygia guttata*, and common whitethroat, *Sylvia communis*) using microarray technology. In both species, the male-bias in gene expression was significantly higher for Z than for autosomes, although the ratio of Z-linked to autosomal expression (Z:A) was relatively close to one in both sexes (range: 0.89–1.01). Interestingly, the Z-linked male-bias in gene expression increased with expression level, and genes with low expression showed the lowest degree of sex-bias. These results support the view that the heterogametic females have up-regulated their single Z-linked homologues to a high extent when the W-chromosome degraded and thereby managed to largely balance their Z:A expression with the exception of highly expressed genes. The male-bias in highly expressed genes points towards male-driven selection on Z-linked loci, and this and other possible hypotheses are discussed.

## Introduction

Many mammals and birds, and several important genetic model organisms, e.g. *Drosophila* spp. and *Caenorhabditis elegans*, have sex chromosomes that differ in gene content. In male heterogametic species (where females are XX and males either XY or X0), the X chromosome holds a large number of functional genes whereas in many species the Y chromosome is degraded due to cessation of recombination, leading to accumulation of deleterious mutations and loss of gene functionality [1]–[4]. Despite unequal sex chromosome copy numbers in males and females, the expression of X-linked genes is not much lower in the heterogametic males than in homogametic females in some species, including in *Drosophila melanogaster*, *C. elegans*, *Anopheles gambiae*, *Tribolium castaneum*, and possibly also in mammals [5]–[13] (see [10], [14] for a contrasting view in mammals). Moreover, in at least some tissues of *Drosophila*, *Anopheles* and mammals, mean expression on the single X chromosome in males is similar to that on autosomes [5]–[7], [10], [12], [13], [15]. The mechanisms resulting in this balance in expression between the sexes, and between sex-linked and autosomal expression, are referred to as dosage compensation [16]–[19]. It is believed that dosage compensation occurs because changes in gene copy number might otherwise have detrimental effects on gene expression in crucial genetic networks [20]–[22]. Dosage compensation was long believed to be essential although recent research in e.g. birds, butterflies, sticklebacks and platypus, clearly show that the sex chromosome to autosomal expression ratios (X:A or Z:A) of less than one can frequently be tolerated in the heterogametic sex [23]–[30] [reviewed in 19].

Sophisticated dosage compensation mechanisms, such as sex chromosome inactivation and hyper-transcription, have obviously evolved to an effective and chromosome-wide state in some male heterogametic systems, such as *Drosophila*, *C. elegans*, *Tribolium* and potentially mammals [5]–[9], [31]–[33] [but see 34], and similar although transient mechanisms have been suggested in both platypus (XY) and chicken (ZW) [27], [35], [36]. This indicates the importance of retaining original expression levels of sex-linked genes in the heterogametic sex as the non-recombining sex chromosome degrades. However, recent genome-wide gene expression studies based on microarray and RNA sequencing data in both male and female heterogametic systems clearly demonstrate an overrepresentation of sex-biased genes on the sex chromosomes, and X:A or Z:A expression ratios of less than one in the heterogametic sex [8], [23]–[30], [37]–[41] [reviewed in 19]. This shows that complete dosage compensation is not an obligatory by-product of sex chromosome degradation and suggests that in several organisms the heterogametic sex either is not strongly negatively affected by inefficient dosage compensation or achieves sufficient dosage compensation without up-regulating the expression of their X- or Z-linked genes to the same levels as in the homogametic sex [18], [19], [42], [43]. To understand the complexity and evolution of sex-biased gene expression and dosage compensation mechanisms, additional gene expression data analysed from novel perspectives are clearly needed [10], [11], [18], [19], [42]–[44]. This is important since sex-biased gene expression has wide-ranging effect on the phenotype and is expected to be tightly linked to reproduction through natural and sexual selection [19], [45]–[55].

We have previously studied the extent of sex-biased gene expression in two passerine birds, the zebra finch (*Taeniopygia guttata*) and the common whitethroat (*Sylvia communis*) [29]. These two species share a common ancestor approximately 24–51 million years ago [56], [57]. We found that genes with male-biased gene expression were overrepresented on the Z chromosome when analysing brain tissue of the zebra finch and the common whitethroat [29]. A high proportion, 90%, of the 509 ESTs that were significantly sex-biased in the zebra finch, and 92% of the 345 ESTs that were sex biased in the common whitethroat, were Z-linked and male-biased [29]. Similar patterns have been described also in other zebra finch expression studies [25], [26] as well as in chicken [24], [39] and European crow *Corvus corone* [30].

In the present study, we address the potential association between overall gene expression and degree of sex-biased gene expression. The evolution of sex-biased gene expression is expected as a result of accumulation of sexually antagonistic mutations, and the outcome depends on the degree of dominance of the alleles and which of the two sexes benefits [58]–[60]. Sex-biased expression of sex-linked genes is expected to evolve when the heterogametic sex supresses its expression as a response to the accumulation of dominant homogametic-advantageous/heterogametic-harmful mutations on Z (or X). Recessive mutations beneficial to the heterogametic sex may also accumulate, but will be expressed in the homogametic sex only when they have reached higher frequencies and so selection for down-regulation should be weaker compared to the case of dominant homogametic-advantageous/heterogametic-harmful mutations. From this follows the expectation that sex-biased genes should have lower levels of average expression than unbiased ones [58]. Connallon and Knowles [59] tested this hypothesis by comparing microarray expression data for male-biased, female-biased, and unbiased genes in *Drosophila*. Surprisingly, they found that sex-biased genes were on average transcribed at higher rates than unbiased genes [59] [see also 61], and similar patterns were recently detected in zebra finch [26] and European crow [30]. We quantify expression on Z-linked and autosomal genes in the brain of zebra finches and common whitethroats using microarrays spotted with genome-wide zebra finch ESTs [29], [62]. Our main finding is a pronounced positive association between the degree of male-bias and overall expression of Z-linked genes in both species. We discuss this in the light of hypotheses of the evolution of sex-biased gene expression [19], [43], [58], [63].

## Materials and Methods

We have analyzed microarray gene expression data from hybridizations of brain tissue from 12 adult zebra finches (6 males and 6 females) and 22 adult common whitethroats (11 males and 11 females). The correct ethical approvals for conducting the experiments were obtained from Malmö/Lund’s ethical committee (“Malmö/Lund djurförsöksetiska nämnd”; www.sjv.se/amnesomraden/djur/forsoksdjur/etiskprovning). Birds were in good condition and were sacrificed through decapitation to ameliorate suffering. For detailed information regarding sample handling, quality control results, hybridization and initial analysis of data, including normalization and filtering, see Naurin *et al*. [29]. Background correction in these expression data sets is the same as in Naurin *et al*. [29] in all aspects but one; in the present analyses we have also removed the 2% of ESTs with the lowest signals. This is in line with Affymetrix’s standard approach where the 2% lowest probes are removed (which for standard arrays is performed before any background correction according to hybridization on mis-match probes). This was done in order to avoid including ESTs with signals close to the background level in the analyses.

### The Lund-zfa Array

The Lund-zfa array is an Affymetrix custom array that consists of 22360 zebra finch ESTs, representing approximately 15800 genes [62], [64]. All ESTs have been BLASTed against the 3.2.4 build of the zebra finch genome [29], [41] and hits in that BLAST was considered significant if the E-value was ≤10^−20^. In total, 1104 of the ESTs with intensities higher than the background cutoff in our data set have significant hits against the Z chromosome and 18520 ESTs have significant autosomal hits. Out of these significantly annotated ESTs, 22 were identified as Z-linked and female-biased in a previous study of sexual dimorphism in gene expression [29]. These ESTs had lower identities with the Z chromosome than male-biased ESTs (90% for female biased ESTs and 99% for male-biased ESTs) and three of them had significant hits against the W chromosome in chicken (*Gallus gallus*) [29]. In order to avoid analyzing potential W gene expression, we excluded these ESTs from the present analyses (including these ESTs did not qualitatively affect our results).

### The Common Whitethroat Hybridization Efficiency

The somewhat lower degree of hybridization on microarray probes caused by sequence divergence between whitethroat RNA and the zebra finch-based microarray can be efficiently controlled via filtering (see Naurin *et al*. [29], [62] for description of the comparative genome hybridization, CGH, of the common whitethroat and how data generated from those studies can be used for filtering).

### Analyses of Dosage Compensation

Log_2_ gene expression intensity data for all ESTs with a significant BLAST hit against either an autosome or the Z chromosome were imported into SPSS Statistics 17.0. For each species, we conducted one-way ANOVAs using expression intensities to compare Z-linked versus autosomal ESTs for females and males, respectively, as well as the intensity of Z-linked ESTs in females versus Z-linked intensity in males. Fold Change (FC) was calculated as the mean unlogged male/mean unlogged female expression intensity. Mean FC for Z-linked ESTs versus autosomal ESTs was tested with a one-way ANOVA. Kolmogorov-Smirnov two-sample tests were used in order to test whether or not the distribution of FC was significantly different for Z-linked and autosomal ESTs. All results as listed below remained unchanged when non-parametric tests were used instead of ANOVAs.

General Linear Models (GLM) were used in order to test whether or not male-biased gene expression increased with increasing expression of Z-linked genes. Residuals for all GLMs were normally distributed.

## Results

### Analyses of Dosage Compensation

**Figure 1:**
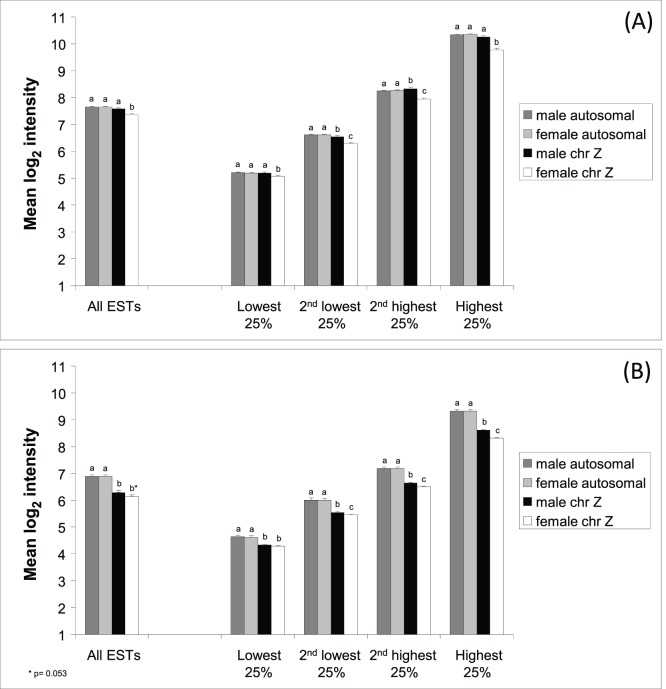
Mean (±SE) gene expression intensity of autosomal and Z-linked ESTs in (A) zebra finch and (B) common whitethroat. Data for all ESTs and when divided into quartiles by mean gene expression intensity from lowest 25% to highest 25% are given. Significant differences (*p*<0.05) between categories are indicated (a, b and c).

**Figure 2:**
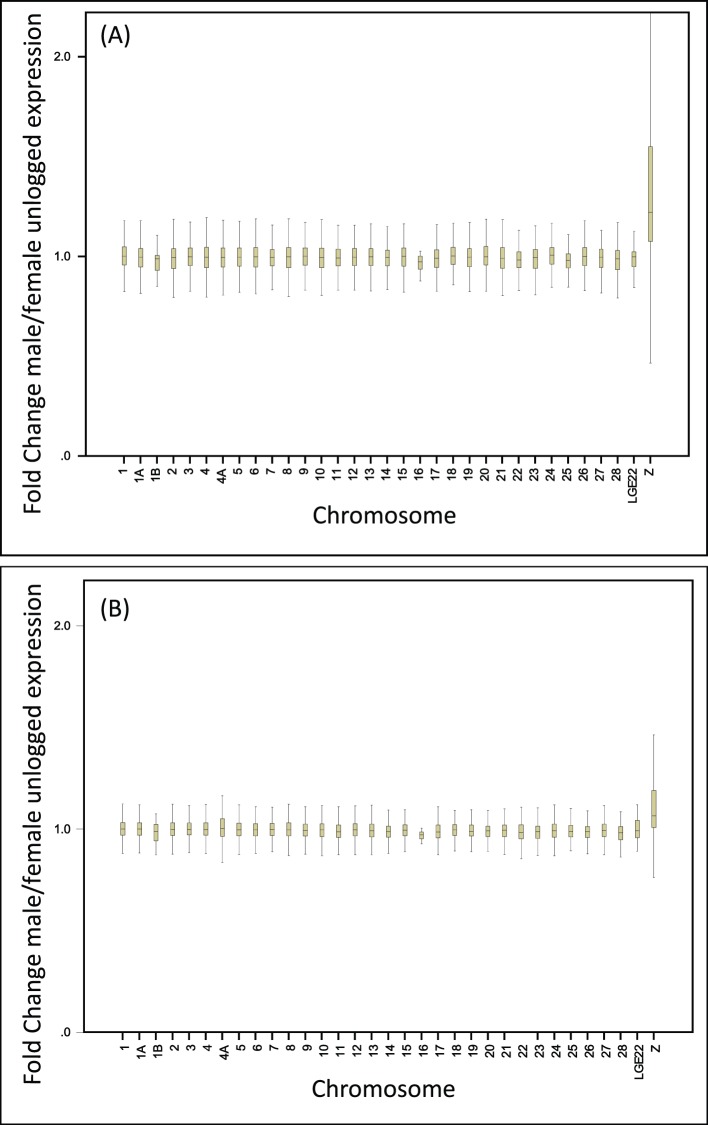
Box plot of Fold Change (i.e. male/female intensity) for individual chromosomes for the zebra finch (A) and the common whitethroat (B).

**Figure 3:**
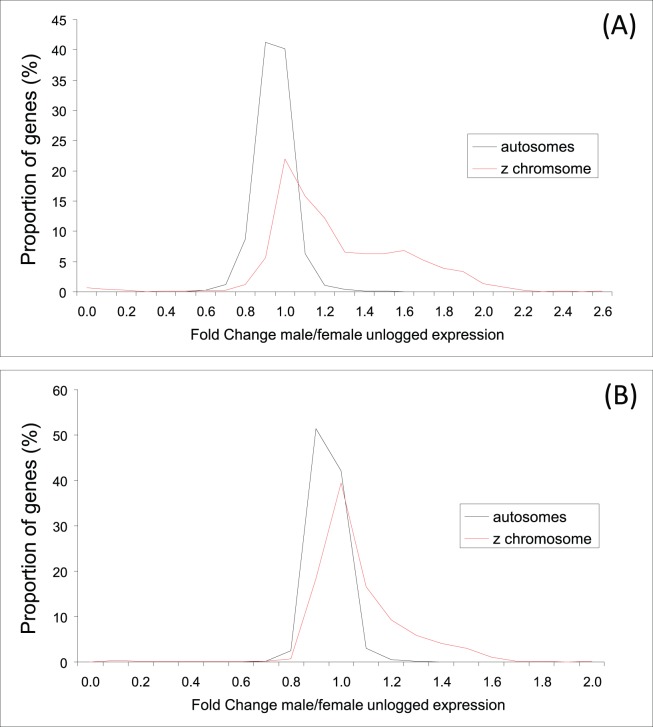
Distributions of Fold Change (i.e. male/female intensity) for (A) the zebra finch and (B) the common whitethroat.

In the zebra finch, Z-linked gene expression was on average significantly lower in females (mean ± SE: 7.26±0.056) than in males (7.57±0.060; F_1, 2208_ = 14.2, p<0.0001) (Figure 1A). Moreover, gene expression in females was significantly lower for Z-linked genes (7.26±0.056) than autosomal genes (7.66±0.015; F_1, 19623_ = 42.9, p<0.0001) (Figure 1A). Male Z-linked expression (7.57±0.060) was not significantly different from autosomal expression (7.65±0.014; F_1, 19623_ = 1.72, p = 0.192). Fold Change (FC; i.e. male/female intensity) was significantly higher for Z (1.31±0.01) than for autosomes (0.997±0.001; F_1, 19623_ = 6686, p<0.0001; Figure 2A), and the distribution of FC-values was significantly different for Z-linked and autosomal ESTs (p<0.0001; Kolmogorov-Smirnov two-sample test; Figure 3A).

For the common whitethroat, Z-linked gene expression tended to be lower in females (6.14±0.048) than in males (6.28±0.050; F_1, 2208_ = 3.73, p = 0.053) (Figure 1B). As in the zebra finch, female intensity in the common whitethroat was significantly lower for Z-linked (6.14±0.048) than autosomal genes (6.90±0.013; F_1, 19623_ = 181.4, p<0.0001) (Figure 1B). Male Z-linked intensity in the common whitethroat (6.28±0.050) was also significantly lower than autosomal expression (6.89±0.013; F_1, 19623_ = 120, p<0.0001; Figure 1B); a pattern that is different from that found in the zebra finch. Fold Change (FC) was significantly higher for Z (1.12±0.005) than for autosomes (0.998±0.001; F_1, 19623_ = 2809, p<0.0001; Figure 2B). The distribution of FC for Z-linked genes was significantly different from the autosomal distribution (p<0.0001; Kolmogorov-Smirnov two-sample test; Figure 3B).

### Association between Male-biased Gene Expression and Overall Expression Level

**Figure 4:**
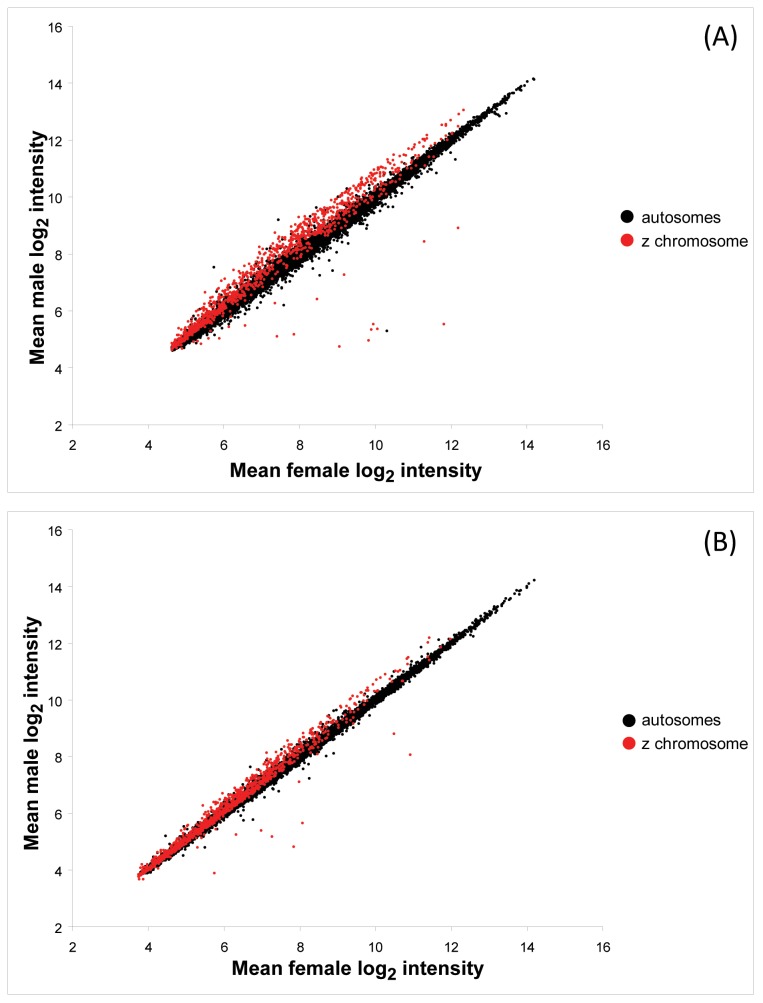
Male gene expression intensity plotted against female expression intensity in (A) the zebra finch and (B) the common whitethroat.

We tested how the male-bias in gene expression on the Z chromosome was related to the general gene expression level. The degree of male-bias in expression of Z-linked genes increased with increasing mean gene expression levels and this pattern was significant in both species (Figures 1 and 4). In GLMs with male intensity as dependent variable, and female intensity, chromosome type (Z or autosomal) and the interaction term as independent variables, the interaction term was highly significant (zebra finch: p<0.0001, Table 1; common whitethroat: p<0.0001, Table 2). In other words, male Z-linked expression increased more steeply with female gene expression than did male autosomal expression (Figure 4). All results from GLMs remained significant even if the 25% of the dataset with the lowest expression was removed, and the results are therefore not affected by detection rates of the microarray. Linear regressions between male and female expression levels showed that Z-linked expression had steeper regression slopes (higher β coefficient) than autosomal expression in both species (zebra finch: β_Z-linked_ = 1.068±0.005, β_autosomal_ = 0.992±0.001; common whitethroat: β_Z-linked_ = 1.053±0.004, β_autosomal_ = 0.998±<0.0001) (Figure 4).

**Table 1:** Results from a General Linear Model with male expression intensity as dependent variable, and female expression intensity, chromosome type (Z or autosomal) and their interaction term as independent variables in the zebra finch.

Source	Type III Sum of Squares	df	Mean Square	F	Sig.	Observed Power^b^
Corrected Model	46413.6^a^	3	15471.2	626361.2	<0.001	1.00
Intercept	0.3	1	0.3	11.2	0.001	0.92
Female log2 intensity	14568.5	1	14568.5	589815.6	<0.001	1.00
Chromosome type	1.8	1	1.8	73.7	<0.001	1.00
Chrom. type * Female log2 int.	19.9	1	19.9	807.6	<0.001	1.00
Error	284.4	11515	0.0			
Total	712863.3	11519				
Corrected Total	46698.0	11518				

a R^2^ = 0.994 (Adjusted R^2^ = 0.994).

b Computed using alpha = 0.05.

**Table 2:** Results from a General Linear Model with male expression intensity as dependent variable, and female expression intensity, chromosome type (Z or autosomal) and their interaction term as independent variables in the common whitethroat.

Source	Type III Sum of Squares	df	Mean Square	F	Sig.	Observed Power^b^
Corrected Model	39293.4^a^	3	13097.8	1310155.4	<0.001	1.00
Intercept	1.7	1	1.7	170.4	<0.001	1.00
Female log2 intensity	10846.4	1	10846.4	1084952.2	<0.001	1.00
Chromosome type	2.4	1	2.4	240.7	<0.001	1.00
Chrom. type * Female log2 int.	8.0	1	8.0	797.8	<0.001	1.00
Error	115.2	11528	0.0			
Total	563563.7	11532				
Corrected Total	39408.6	11531				

a R^2^ = 0.994 (Adjusted R^2^ = 0.994).

b Computed using alpha = 0.05.

### Z-linked in Relation to Autosomal Gene Expression

In order to evaluate how the pattern of gene expression on the Z chromosome relates to autosomal gene expression, we divided the Z-linked and the autosomal dataset into four equal parts (quartiles) by sorting all ESTs by mean log_2_ expression (calculated for all individuals regardless of sex). Each quartile was then analyzed separately (Figure 1).

In the zebra finch, female Z-linked expression was significantly lower than male Z-linked and autosomal expression in all four categories (p<0.0001; Table 3). Male Z-linked expression was slightly (but significantly) lower than mean autosomal expression in the quartile with the second lowest expression (p = 0.013), while slightly higher in the quartile with the second highest expression (p = 0.008; Table 3). Overall, male gene expression closely followed that of the autosomes, while female expression was close to autosomal expression only in the quartile with lowest gene expression and then became progressively lower with higher mean expression in comparison to both male Z-linked and autosomal genes (Figure 1A). The mean Z-linked over mean autosomal expression ratios (Z:A ratios) for the four quartiles of gene expression ranged between 0.98 and 0.94 (with the lowest ratio in the high quartile) in females and between 0.99 and 1.01 in males.

**Table 3:** Gene expression intensities in all four quartiles in the zebra finch.

	female Z	male Z	female autosomes	male autosomes
*lowest 25%*				
mean log2 expression	5.07	5.18	5.19	5.2
standard error	0.02	0.022	0.008	0.008
*female Z vs. male Z: F = 14.3; P<0.0001*				
*female Z vs. autosomes: F = 24.6; P<0.0001*				
*male Z vs. autosomes: F = 1.06; P = 0.304*				
*2nd lowest 25%*				
mean log2 expression	6.28	6.54	6.62	6.62
standard error	0.029	0.033	0.009	0.009
*female Z vs. male Z: F = 33.3; P<0.0001*				
*female Z vs. autosomes: F = 127.9; P<0.0001*				
*male Z vs. autosomes: F = 6.24; P = 0.013*				
*2nd highest 25%*				
mean log2 expression	7.93	8.33	8.27	8.24
standard error	0.036	0.041	0.009	0.009
*female Z vs. male Z: F = 56.5; P<0.0001*				
*female Z vs. autosomes: F = 114.2; P<0.0001*				
*male Z vs. autosomes: F = 7.03; P = 0.008*				
*highest 25%*				
mean log2 expression	9.76	10.3	10.4	10.3
standard error	0.054	0.054	0.019	0.019
*female Z vs. male Z: F = 40.2; P<0.0001*				
*female Z vs. autosomes: F = 94.4; P<0.0001*				
*male Z vs. autosomes: F = 2.41; P = 0.121*				

Also shown are results for one-way ANOVAs of female and male Z-linked and autosomal expression.

In the common whitethroat, Z-linked expression was significantly lower than autosomal expression in both sexes in all four quartiles (p<0.0001; Table 4; Figure 1B). Female Z-linked expression was significantly lower than male Z-linked expression in the three groups with highest expression intensities (p<0.0001 for the highest and second highest and p = 0.002 for the second lowest group) but not significantly different in the lowest quartile (p = 0.223; Table 4; Figure 1B). The Z:A ratios for the four quartiles of gene expression ranged between 0.93 and 0.89 (with the lowest ratio in the high quartile) in females and between 0.93 and 0.92 in males.

**Table 4:** Gene expression intensities in all four quartiles in the common whitethroat.

	female Z	male Z	female autosomes	male autosomes
*lowest 25%*				
mean log2 expression	4.28	4.32	4.62	4.63
standard error	0.023	0.022	0.023	0.01
*female Z vs. male Z: F = 1.48; P = 0.223*				
*female Z vs. autosomes: F = 103.2; P<0.0001*				
*male Z vs. autosomes: F = 80.3; P<0.0001*				
*2nd lowest 25%*				
mean log2 expression	5.47	5.56	6.01	6.01
standard error	0.019	0.02	0.006	0.006
*female Z vs. male Z: F = 9.91; P = 0.002*				
*female Z vs. autosomes: F = 697.5; P<0.0001*				
*male Z vs. autosomes: F = 495.7; P<0.0001*				
*2nd highest 25%*				
mean log2 expression	6.52	6.65	7.19	7.19
standard error	0.043	0.044	0.013	0.013
*female Z vs. male Z: F = 16.6; P<0.0001*				
*female Z vs. autosomes: F = 756.5; P<0.0001*				
*male Z vs. autosomes: F = 482.6; P<0.0001*				
*highest 25%*				
mean log2 expression	8.32	8.6	9.32	9.32
standard error	0.063	0.064	0.023	0.023
*female Z vs. male Z: F = 9.88; P<0.0001*				
*female Z vs. autosomes: F = 179.3; P<0.0001*				
*male Z vs. autosomes: F = 90.3; P<0.0001*				

Also shown are results for one-way ANOVAs of female and male Z-linked and autosomal expression.

### Sequence Divergence in Common Whitethroat

Mean number of significantly hybridizing probes (out of the 11 possible) for Z-linked genes (9.82±0.040) in our cross-species comparative genome hybridization (CGH) study [62] was significantly lower than mean number of hybridizing probes on autosomes (10.23±0.009; F_1, 19615_, p<0.0001). This result suggests a high rate of sequence evolution on Z. Thus, our common whitethroat expression data might be affected by the somewhat higher degree of sequence divergence on the Z chromosome than on the autosomes. We therefore re-analyzed the data set for the common whitethroat, this time using only ESTs that had significant hybridization on all of the 11 probes for each EST on the array in the previous CGH study [62]. This data set included 10370 autosomal and 443 Z-linked ESTs. For this restricted data set the results remained quantitatively unchanged. However, as expected if Z evolves faster than the autosomes, Z-linked expression was more affected by the removal of ESTs with some degree of sequence divergence (female Z-linked intensity was on average 8.4% higher and male Z-linked intensity 8.6% higher, while the autosomal expression intensity was only 5.0% higher in both sexes).

## Discussion

Our present results on common whitethroat and zebra finch, together with previous results on chicken, zebra finch and European crow [23]–[26], [29], [30], [39], [65], clearly demonstrate that male-biased gene expression on the avian Z chromosome cannot be explained by a simple effect of the double dose of Z chromosome in males compared to females. Ratios of Z-linked to autosomal gene expression (Z:A) were relatively close to one in both sexes of both species (range: 0.89–1.01) with the lowest ratios for females in the subset (quartile) of highly expressed genes (0.94 in zebra finches and 0.89 in common whitethroat). Previous studies report similar levels for chicken and zebra finch males [23]–[25], but somewhat lower values for zebra finch and European crow females (*c.* 0.80) [26], [30]. These results indicate that in birds the heterogametic females have up-regulated their single Z-linked homologues to a high extent when the W chromosome degraded and have thereby largely kept the balance between Z-linked and autosomal expression. This suggests that female birds achieve dosage compensation to a very high degree, although without chromosome-wide mechanisms [25], [26], [36], [66], [67]. A recent study which analysed level of expression on the current Z and compared it to that of a putative proto-Z came to the same conclusion of partial, but not complete, dosage compensation in female chicken [68]. However, even though females achieve a high degree of compensation there is still extensive and significant male-biased Z-linked gene expression in both our study species. When examining this in more detail, we found the interesting pattern that in both species the male-biased expression of Z-linked genes increased with increasing gene expression level from a more or less sex-balanced expression among Z-linked genes with low expression. This mainly resulted from a more pronounced drop in female Z-linked expression with higher mean expression than was the case for both male Z-linked and autosomal gene expression (see Figure 1). Together with the indications of increasing male-bias with increased gene expression found by Melamed and Arnold [23] in the chicken, and the finding of higher expression of male-biased than unbiased genes recently described in zebra finches and European crow [26], [30], our results from yet another passerine, the common whitethroat, as well as for the zebra finch, indicate that the pattern of lower female expression for highly expressed genes on Z is a general phenomenon among birds.

In addition to those patterns concerning sex-biased gene expression, we found that in the common whitethroat both males and females had lower expression of genes on the sex chromosomes than of those on the autosomes, and this was particularly true for highly expressed genes (Figure 1). This is interesting in relation to the result from our previous CGH study (comparing the number of significantly hybridizing probes on the microarray) that showed high sequence divergence on the Z chromosome between the common whitethroat and the zebra finch [62]; and such patterns are also found in other species [48], [51], [69]. The pronounced drop in Z-linked gene expression for highly expressed genes in both male and female common whitethroats observed here suggests that divergence is particularly pronounced for Z-linked genes with medium to high expression levels. Similar patterns based on data of sequence divergence (dN/dS analyses) between chicken and zebra finch were reported by Itoh *et al*. [26]. The evolution of Z-linked genes is affected by the lower effective population size compared to autosomal genes [70]–[72]. Moreover, Z is implicated in adaptive evolution, perhaps to a larger extent than X in e.g. mammals [71], [73]. The potential role of Z-linked genes in the evolution of male-specific traits and sexual selection makes the high rate of molecular evolution on Z highly interesting [19], [43], [58], [63]. In *Ficedula* flycatchers genes involved in speciation are predominantly Z-linked and coding for sex specific traits, like male plumage characteristics and female species recognition [74]. However, also in some male heterogametic systems such pattern have been found; for example, in *Drosophila*, the X chromosome harbours genes involved in female mate-choice underlying sexual isolation [75] (see also [34], [54], [61], [76]).

### Male-biased Expression of Highly Expressed Genes on Z

Even if it may seem unlikely that the somewhat lower Z-linked than autosomal expression in females should disrupt critical networks, there must be a compelling reason for why females do not balance their Z-linked expression to that of the autosomes for genes with high mean expression while achieving full balance for genes with low expression. We suggest three possible explanations for this:

First, if there was a chromosome-wide mechanism of dosage compensation also in birds a general up-regulation of Z-linked genes along the chromosome may have occurred in females to a degree equal to the level of low to medium expressed genes (but not to the level of highly expressed genes). However, this explanation seems unlikely as the level of compensation is a highly variable process in birds and varies between tissues and age groups in a way that indicates that selection for up-regulation has taken place on a gene to gene basis rather than on a chromosome-wide level [25], [26], [36], [66], [67].

Second, selection for higher expression in females (to compensate for loss of expression as W-linked gene homologue degrades) might cause a correlated response in males, thereby increasing their gene expression as well. If increased expression is detrimental in males, then Z-linked genes might face further selection for down-regulation (causing an opposing spillover effect in females). Such opposing selection pressures could explain why female have not fully balanced all their Z-linked genes. This scenario has been suggested to explain the complex sex-biased gene expression with female overcompensation (X:A>1) in *Tribolium* beetles [8]. However, it is not clear why this should preferentially affect highly expressed genes. Moreover, if males are more sensitive to increased expression in high-expressed than in low-expressed genes, it might be expected that selection in females will affect the male Z:A ratio for lowly expressed genes; a pattern not seen in our data.

Third, some property of many of the highly expressed genes might make them less suitable for high expression in females. It is likely that females have faced selection for up-regulation of Z homologues as W degraded [16]–[21]. However, the two chromosomes would have evolved separately for some time before selection for dosage compensation took place. Male-biased mutation rate [77]–[79] and the potential for high divergence in sex-biased Z-linked genes [43], [70], [80], [81] give rise to obvious questions: Have some of the Z-linked genes evolved to increase male function due to high degree of sexual selection in males; and when females cannot rely on the degrading W any longer, would they even benefit from lower levels of compensation in order to avoid malfunction of these male-adapted (female-detrimental) genes?

Similar results to these now found in birds [23,26,30, this study] were previously found by Connallon and Knowles [59] in *Drosophila*; sex-biased genes were on average expressed at higher rates than unbiased genes [see also 61]. Interestingly, the pattern of male-bias is reported to be reversed in the silkworm (*Bombyx mori*) where the male-bias decreases with increasing gene expression level, creating a pattern where male-biased genes are expressed at lower levels than un-biased genes [40] (however, see [82] for a re-analysis of Zha et al.’s data). Zha et al.’s result in the silkworm – but not the results in birds – is in line with Rice’s [58] original hypothesis suggesting that genes become sex-biased when their expression in the harmed sex is decreased or abolished, and therefore, on average, sex-biased genes should have lower levels of expression than unbiased ones.

It has been suggested that sex-biased genes may be more dispensable [83] – a hypothesis which is intuitively easy to grasp – genes with low fitness effects should be more prone to respond to any kind of selection (sexual or otherwise) and sex-biased genes do show high degrees of sequence evolution in general. However, if this is true, one would expect a pattern like the one in silkworm (where genes with low expression show a high degree of sex-bias [40]) to be the most commonly reported scenario. The fact that the opposite seems to be true is highly interesting given the fact that highly expressed genes have a low degree of sequence evolution and are often assumed to be more essential [44], [61], [70], [76], [84]–[91]. Hence, the patterns of sex-bias in relation to gene expression levels reported to date do not support the idea that simple dispensability is what makes a gene prone to accumulate sex-bias or respond to sexual selection; other mechanisms must also be invoked if we are to understand which parts of the genome are more likely to obtain sex-specific functions.

To explain that highly expressed male-biased genes in *Drosophila* are less frequently located on the X chromosome than are lowly expressed male-biased genes [e.g. 76], Vicoso and Charlesworth [92] proposed the following hypothesis: The increase in expression required to make a gene male-biased is less likely when X-linked because there is an upper limit to the rate of expression that can be achieved [93], [94] and because the X chromosome is often hyper-activated in *Drosophila* males as a result of the evolution of dosage compensation [5]–[8], [31]–[33] [but see 34]. This type of hypothesis is however unlikely to explain the observation of increasing male-biased gene expression with increasing expression in female heterogametic birds, because there seem to be no chromosome-wide dosage compensation [24]–[26], [36], [66], [67]. However, the idea that there may be an upper level to the rate of expression that can be achieved with a single gene copy, in this case on the Z in the hemizygous sex, due to space limitations on the DNA strand for binding transcriptional molecules, could explain why females may have difficulties in fully balancing the most highly expressed Z-linked genes. However, we find this suggestion somewhat less likely because the sex-biased Z-linked genes are not among the highest expressed in our data set, thus do not seem saturated (Figure 4), and because it remains unclear why such an expression ceiling would apply only to some species (e.g. this study) and not others [5], [6], [10], [12], [13], [15], [40].

The result that male-biased and unbiased genes in chicken are not only associated with certain expression levels but also have functional differences [23], and that female detrimental antagonism is overrepresented on the Z chromosome [95], would imply that selection on Z-linked genes in males have adapted them to male function in a way that makes them unsuitable for up-regulation in females [43], [55]. However, observations of such functional differences and accumulation of female detrimental antagonisms do not provide an explicit hypothesis to explain the positive association between male-biased Z-linked expression and overall gene expression now found in birds. Moreover, the data available for autosomal genes from several taxa suggest that sex-biased genes evolve fast, whereas highly expressed genes – as mentioned above – in general have a low degree of sequence, protein and expression profile evolution [44], [61], [70], [76], [84]–[91]. Hence, it is surprising to find a proportionally large amount of male-bias in highly expressed Z-linked genes in both the chicken, crow, zebra finch and the common whitethroat, a finding that suggests that there has been a high degree of sex-specific selection at Z-linked loci in birds and therefore that highly expressed Z-linked genes are hotspots for evolution of sexual dimorphism in birds [19], [34], [43], [58], [63], [73], [74], [91], [96]. Further studies of gene expression and sexual antagonism in both female and male heterogametic systems are clearly needed to elucidate patterns and processes of sex-biased gene expression on the sex chromosomes [cf. e.g. 19,44].

## References

[1] Charlesworth B, Charlesworth D (2000) The degeneration of Y chromosomes. Philos Trans R Soc Lond B 355: 1563–1572. 11127901 10.1098/rstb.2000.0717 PMC1692900

[2] Charlesworth D, Charlesworth B, Marais G (2005) Steps in the evolution of heteromorphic sex chromosomes. Heredity 95: 118–128. 15931241 10.1038/sj.hdy.6800697

[3] Mank JE, Ellegren H (2007) Parallel divergence and degradation of the avian W sex chromosome. Trends Ecol Evol 22: 389–391. 17573147 10.1016/j.tree.2007.05.003

[4] Bergero R, Charlesworth D (2009) The evolution of restricted recombination in sex chromosomes. Trends Ecol Evol 24: 94–102. 19100654 10.1016/j.tree.2008.09.010

[5] Hahn MW, Lanzaro GC (2005) Female-biased gene expression in the malaria mosquito *Anopheles gambiae* . Curr Biol 15: R192–R193. 15797007 10.1016/j.cub.2005.03.005

[6] Nguyen DK, Disteche CM (2006) Dosage compensation of the active X chromosome in mammals. Nat Genet 38: 47–53. 16341221 10.1038/ng1705

[7] Lin H, Gupta V, Vermilyea MD, Falciani F, Lee JT, et al (2007) Dosage compensation in the mouse balances up-regulation and silencing of X-linked genes. PLoS Biol 5: e326. 18076287 10.1371/journal.pbio.0050326 PMC2121114

[8] Prince EG, Kirkland D, Demuth JP (2010) Hyperexpression of the X chromosome in both sexes results in extensive female bias of X-linked genes in the flour beetle. Genome Biol Evol 2: 336–346. 20624738 10.1093/gbe/evq024 PMC2942036

[9] Larschan E, Bishop EP, Kharchenko PV, Core LJ, Lis JT, et al (2011) X chromosome dosage compensation via enhanced transcriptional elongation in Drosophila. Nature 471: 115–118. 21368835 10.1038/nature09757 PMC3076316

[10] Xiong Y, Chen X, Chen Z, Wang X, Shi S, et al (2010) RNA sequencing shows no dosage compensation of the active X-chromosome. Nat Genet 42: 1043–1047. 21102464 10.1038/ng.711

[11] Deng X, Hiatt JB, Nguyen DK, Ercan S, Sturgill D, et al (2011) Evidence for compensatory upregulation of expressed X-linked genes in mammals, *Caenorhabditis elegans* and *Drosophila melanogaster* . Nat Genet 43: 1179–1185. 22019781 10.1038/ng.948 PMC3576853

[12] Lin H, Halsall JA, Antczak P, O'Neill LP, Falciani F, et al (2011) Relative overexpression of X-linked genes in mouse embryonic stem cells is consistent with Ohno's hypothesis. Nat Genet 43: 1169–1170. 22120049 10.1038/ng.992

[13] Kharchenko PV, Xi R, Park PJ (2011) Evidence for dosage compensation between the X chromosome and autosomes in mammals. Nat Genet 43: 1167–1169. 22120048 10.1038/ng.991

[14] Reinius B, Shi C, Hengshuo L, Sandhu KS, Radomska KJ, et al (2010) Female-biased expression of long non-coding RNAs in domains that escape X-inactivation in mouse. BMC Genomics 11: 614. 21047393 10.1186/1471-2164-11-614 PMC3091755

[15] Baker DA, Russell S (2011) Role of testis-specific gene expression in sex-chromosome evolution of *Anopheles gambiae* . Genetics 189: 1117–1120. 21890740 10.1534/genetics.111.133157 PMC3213352

[16] Ohno S (1967) Sex chromosomes and sex-linked genes. Springer, Berlin.

[17] Lucchesi JC, Kelly WG, Parming B (2005) Chromatin remodeling in dosage compensation. Ann Rev Gen 39: 615–651. 10.1146/annurev.genet.39.073003.094210 16285873

[18] Straub T, Becker PB (2007) Dosage compensation: the beginning and end of generalization. Nat Rev Genet 8: 47–57. 17173057 10.1038/nrg2013

[19] Mank JE, Hosken DJ, Wedell N (2011) Some inconvenient truths about sex chromosome dosage compensation and the potential role of sexual conflict. Evolution 65: 2133–2144. 21790564 10.1111/j.1558-5646.2011.01316.x

[20] Lindsley DL, Sandler L, Jacobs PA, Nozawa H, Parry DM, et al.. (1972) Segmental aneuploidy and genetic gross structure of *Drosophila* genome. Genetics 71: 157–&. 10.1093/genetics/71.1.157 PMC1212769 4624779

[21] Rosenbusch B (2004) The incidence of aneuploidy in human oocytes assessed by conventional cytogenetic analysis. Hereditas 141: 97–105. 15660969 10.1111/j.1601-5223.2004.01803.x

[22] Lynch M (2007) The evolution of genetic networks by non-adaptive processes. Nat Rev Genet 8: 803–813. 17878896 10.1038/nrg2192

[23] Melamed E, Arnold AP (2007) Regional differences in dosage compensation on the chicken Z chromosome. Genome Biol 8: R202. 17900367 10.1186/gb-2007-8-9-r202 PMC2375040

[24] Ellegren H, Hultin-Rosenberg L, Brunstrom B, Dencker L, Kultima K, et al (2007) Faced with inequality: chicken do not have a general dosage compensation of sex-linked genes. BMC Biol 5: 40. 17883843 10.1186/1741-7007-5-40 PMC2099419

[25] Itoh Y, Melamed E, Yang X, Kampf K, Wang S, et al (2007) Dosage compensation is less effective in birds than in mammals. J Biol 6: 2. 17352797 10.1186/jbiol53 PMC2373894

[26] Itoh Y, Replogle K, Kim YH, Wade J, Clayton DF, et al (2010) Sex bias and dosage compensation in the zebra finch versus chicken genomes: general and specialized patterns among birds. Genome Res 20: 512–518. 20357053 10.1101/gr.102343.109 PMC2847754

[27] Deakin JE, Hore TA, Koina E, Marshall Graves JA (2008) The status of dosage compensation in the multiple X chromosomes of the platypus. PLoS Genet 4: e1000140. 18654631 10.1371/journal.pgen.1000140 PMC2453332

[28] Leder EH, Cano JM, Leinonen T, O'Hara RB, Nikinmaa M, et al (2010) Female-biased expression on the X chromosome as a key step in sex chromosome evolution in threespine sticklebacks. Mol Biol Evol 27: 1495–1503. 20142438 10.1093/molbev/msq031

[29] Naurin S, Hansson B, Hasselquist D, Kim Y-H, Bensch S (2011) The sex-biased brain: sexual dimorphism in gene-expression in two species of songbirds. BMC Genomics 12: 37. 21235773 10.1186/1471-2164-12-37 PMC3036617

[30] Wolf JB, Bryk J (2011) General lack of global dosage compensation in ZZ/ZW systems? Broadening the perspective with RNA-seq. BMC Genomics 12: 91. 21284834 10.1186/1471-2164-12-91 PMC3040151

[31] Lyon MF (1961) Gene action in the X-chromosome of the mouse (*Mus musculus* L.). Nature 190: 372–373. 13764598 10.1038/190372a0

[32] Baker BS, Gorman M, Marin I (1994) Dosage compensation in *Drosophila* . Annu Rev Genet 28: 491–521. 7893138 10.1146/annurev.ge.28.120194.002423

[33] Charlesworth B (1996) The evolution of chromosomal sex determination and dosage compensation. Curr Biol 6: 149–162. 8673462 10.1016/s0960-9822(02)00448-7

[34] Meiklejohn CD, Landeen EL, Cook JM, Kingan SB, Presgraves DC (2011) Sex chromosome-specific regulation in the Drosophila male germline but little evidence for chromosomal dosage compensation or meiotic inactivation. PLoS Biol 9: e1001126. 21857805 10.1371/journal.pbio.1001126 PMC3156688

[35] Deakin JE, Chaumeil J, Hore TA, Marshall Graves JA (2009) Unravelling the evolutionary origins of X chromosome inactivation in mammals: insights from marsupials and monotremes. Chromosome Res 17: 671–685. 19802707 10.1007/s10577-009-9058-6

[36] Schoenmakers S, Wassenaar E, Hoogerbrugge JW, Laven JS, Grootegoed JA, et al (2009) Female meiotic sex chromosome inactivation in chicken. PLoS Genet 5: e1000466. 19461881 10.1371/journal.pgen.1000466 PMC2678266

[37] Khil PP, Smirnova NA, Romanienko PJ, Camerini-Otero RD (2004) The mouse X chromosome is enriched for sex-biased genes not subject to selection by meiotic sex chromosome inactivation. Nat Genet 36: 642–646. 15156144 10.1038/ng1368

[38] Reinke V, Gil IS, Ward S, Kazmer K (2004) Genome-wide germline-enriched and sex-biased expression profiles in *Caenorhabditis elegans* . Development 131: 311–323. 14668411 10.1242/dev.00914

[39] Kaiser VB, Ellegren H (2006) Nonrandom distribution of genes with sex-biased expression in the chicken genome. Evolution Int J Org Evolution 60: 1945–1951. 17089978

[40] Zha X, Xia Q, Duan J, Wang C, He N, et al (2009) Dosage analysis of Z chromosome genes using microarray in silkworm, *Bombyx mori* . Insect Biochem Mol Biol 39: 315–321. 19150406 10.1016/j.ibmb.2008.12.003

[41] Warren WC, Clayton DF, Ellegren H, Arnold AP, Hillier LW, et al (2010) The genome of a songbird. Nature 464: 757–762. 20360741 10.1038/nature08819 PMC3187626

[42] Mank JE (2009) The W, X, Y and Z of sex-chromosome dosage compensation. Trends Genet 25: 226–233. 19359064 10.1016/j.tig.2009.03.005 PMC2923031

[43] Naurin S, Hansson B, Bensch S, Hasselquist D (2010) Why does dosage compensation differ between XY and ZW taxa? Trends Genet 26: 15–20. 19963300 10.1016/j.tig.2009.11.006

[44] Meisel RP (2011) Towards a more nuanced understanding of the relationship between sex-biased gene expression and rates of protein-coding sequence evolution. Mol Biol Evol 28: 1893–1900. 21239389 10.1093/molbev/msr010 PMC3098513

[45] Swanson WJ, Vacquier VD (2002) The rapid evolution of reproductive proteins. Nat Rev Genet 3: 137–144. 11836507 10.1038/nrg733

[46] Good JM, Nachman MW (2005) Rates of protein evolution are positively correlated with developmental timing of expression during mouse spermatogenesis. Mol Biol Evol 22: 1044–1052. 15647515 10.1093/molbev/msi087

[47] Jagadeeshan S, Singh RS (2005) Rapidly evolving genes of *Drosophila*: Differing levels of selective pressure in testis, ovary, and head tissues between sibling. Mol Biol Evol 22: 1793–1801. 15917496 10.1093/molbev/msi175

[48] Khaitovich P, Hellmann I, Enard W, Nowick K, Leinweber M, et al (2005) Parallel patterns of evolution in the genomes and transcriptomes of humans and chimpanzees. Science 309: 1850–1854. 16141373 10.1126/science.1108296

[49] Proschel M, Zhang Z, Parsch J (2006) Widespread adaptive evolution of *Drosophila* genes with sex-biased expression. Genetics 174: 893–900. 16951084 10.1534/genetics.106.058008 PMC1602082

[50] Torgerson DG, Kulathinal RJ, Singh RS (2002) Mammalian sperm proteins are rapidly evolving: Evidence of positive selection in functionally diverse genes. Mol Biol Evol 19: 1973–1980. 12411606 10.1093/oxfordjournals.molbev.a004021

[51] Ellegren H, Parsch J (2007) The evolution of sex-biased genes and sex-biased gene expression. Nat Rev Genet 8: 689–698. 17680007 10.1038/nrg2167

[52] Rinn JL, Snyder M (2005) Sexual dimorphism in mammalian gene expression. Trends Genet 21: 298–305. 15851067 10.1016/j.tig.2005.03.005

[53] Connallon T, Cox RM, Calsbeek R (2010) Fitness consequences of sex-specific selection. Evolution 64: 1671–1682. 20050912 10.1111/j.1558-5646.2009.00934.x

[54] Innocenti P, Morrow EH (2010) The sexually antagonistic genes of *Drosophila* melanogaster. PLoS Biol 8: e1000335. 20305719 10.1371/journal.pbio.1000335 PMC2838750

[55] Connallon T, Clark AG (2011) Association between sex-biased gene expression and mutations with sex-specific phenotypic consequences in *Drosophila* . Genome Biol Evol 3: 151–155. 21292631 10.1093/gbe/evr004 PMC3048362

[56] Jonsson KA, Fjeldsa J (2006) Determining biogeographical patterns of dispersal and diversification in oscine passerine birds in Australia, Southeast Asia and Africa. J Biogeogr 33: 1155–1165.

[57] Barker FK, Cibois A, Schikler P, Feinstein J, Cracraft J (2004) Phylogeny and diversification of the largest avian radiation. Proc Natl Acad Sci U S A 101: 11040–11045. 15263073 10.1073/pnas.0401892101 PMC503738

[58] Rice WR (1984) Sex chromosomes and the evolution of sexual dimorphism. Evolution 38: 735–742. 28555827 10.1111/j.1558-5646.1984.tb00346.x

[59] Connallon T, Knowles LL (2005) Intergenomic conflict revealed by patterns of sex-biased gene expression. Trends Genet 21: 495–499. 16039005 10.1016/j.tig.2005.07.006

[60] Patten MM, Haig D (2009) Maintenance or loss of genetic variation under sexual and parental antagonism at a sex-linked locus. Evolution 63: 2888–2895. 19573084 10.1111/j.1558-5646.2009.00764.x

[61] Zhang Y, Sturgill D, Parisi M, Kumar S, Oliver B (2007) Constraint and turnover in sex-biased gene expression in the genus *Drosophila* . Nature 450: 233–237. 17994089 10.1038/nature06323 PMC2386141

[62] Naurin S, Bensch S, Hansson B, Johansson T, Clayton DF, et al (2008) A microarray for large-scale genomic and transcriptional analyses of the zebra finch (*Taeniopygia guttata*) and other passerines. Mol Ecol Res 8: 275–281. 10.1111/j.1471-8286.2007.01979.x 21585769

[63] Connallon T, Clark AG (2010) Sex linkage, sex-specific selection, and the role of recombination in the evolution of sexually dimorphic gene expression. Evolution 64: 3417–3442. 20874735 10.1111/j.1558-5646.2010.01136.x PMC2998557

[64] Replogle K, Arnold AP, Ball GF, Band M, Bensch S, et al (2008) The Songbird Neurogenomics (SoNG) Initiative: community-based tools and strategies for study of brain gene function and evolution. BMC Genomics 9: 131. 18366674 10.1186/1471-2164-9-131 PMC2329646

[65] Mank JE, Hultin-Rosenberg L, Webster MT, Ellegren H (2008) The unique genomic properties of sex-biased genes: insights from avian microarray data. BMC Genomics 9: 148. 18377635 10.1186/1471-2164-9-148 PMC2294128

[66] Mank JE, Ellegren H (2009) All dosage compensation is local: gene-by-gene regulation of sex-biased expression on the chicken Z chromosome. Heredity 102: 312–320. 18985062 10.1038/hdy.2008.116

[67] Schoenmakers S, Wassenaar E, Laven JS, Grootegoed JA, Baarends WM (2010) Meiotic silencing and fragmentation of the male germline restricted chromosome in zebra finch. Chromosoma 119: 311–324. 20162291 10.1007/s00412-010-0258-9 PMC2875885

[68] Julien P, Brawand D, Soumillon M, Necsulea A, Liechti A, et al (2012) Mechanisms and evolutionary patterns of Mammalian and avian dosage compensation. PLoS Biol 10: e1001328. 22615540 10.1371/journal.pbio.1001328 PMC3352821

[69] Richards S, Liu Y, Bettencourt BR, Hradecky P, Letovsky S, et al (2005) Comparative genome sequencing of Drosophila pseudoobscura: chromosomal, gene, and cis-element evolution. Genome Res 15: 1–18. 15632085 10.1101/gr.3059305.125 PMC540289

[70] Mank JE, Axelsson E, Ellegren H (2007) Fast-X on the Z: rapid evolution of sex-linked genes in birds. Genome Res 17: 618–624. 17416747 10.1101/gr.6031907 PMC1855182

[71] Ellegren H (2008) Genomic evidence for a large-Z effect. Proc R Soc Lond B 276: 361–366. 10.1098/rspb.2008.1135 PMC2674357 18826931

[72] Mank JE, Nam K, Ellegren H (2010) Faster-Z evolution is predominantly due to genetic drift. Mol Biol Evol 27: 661–670. 19926635 10.1093/molbev/msp282

[73] Qvarnström A, Bailey RI (2009) Speciation through evolution of sex-linked genes. Heredity 102: 4–15. 18781167 10.1038/hdy.2008.93

[74] Saether SA, Saetre GP, Borge T, Wiley C, Svedin N, et al (2007) Sex chromosome-linked species recognition and evolution of reproductive isolation in flycatchers. Science 318: 95–97. 17916732 10.1126/science.1141506

[75] Bailey RI, Innocenti P, Morrow EH, Friberg U, Qvarnstrom A (2011) Female *Drosophila melanogaster* gene expression and mate choice: the X chromosome harbours candidate genes underlying sexual isolation. PLoS One 6: e17358. 21386982 10.1371/journal.pone.0017358 PMC3046225

[76] Ranz JM, Castillo-Davis CI, Meiklejohn CD, Hartl DL (2003) Sex-dependent gene expression and evolution of the *Drosophila* transcriptome. Science 300: 1742–1745. 12805547 10.1126/science.1085881

[77] Ellegren H, Fridolfsson A-K (1997) Male-driven evolution of DNA sequences in birds. Nature Genet 17: 182–184. 9326938 10.1038/ng1097-182

[78] Makova KD, Li WH (2002) Strong male-driven evolution of DNA sequences in humans and apes. Nature 416: 624–626. 11948348 10.1038/416624a

[79] Axelsson E, Smith NG, Sundstrom H, Berlin S, Ellegren H (2004) Male-biased mutation rate and divergence in autosomal, Z-linked and W-linked introns of chicken and Turkey. Mol Biol Evol 21: 1538–1547. 15140948 10.1093/molbev/msh157

[80] Vicoso B, Charlesworth B (2006) Evolution on the X chromosome: unusual patterns and processes. Nat Rev Genet 7: 645–653. 16847464 10.1038/nrg1914

[81] Presgraves DC (2008) Sex chromosomes and speciation in *Drosophila* . Trends Genet 24: 336–343. 18514967 10.1016/j.tig.2008.04.007 PMC2819171

[82] Walters JR, Hardcastle TJ (2011) Getting a full dose? Reconsidering sex chromosome dosage compensation in the silkworm, *Bombyx mori* . Genome Biol Evol 3: 491–504. 21508430 10.1093/gbe/evr036 PMC3296447

[83] Mank JE, Ellegren H (2009) Are sex-biased genes more dispensable? Biol Lett 5: 409–412. 19433612 10.1098/rsbl.2008.0732 PMC2679909

[84] Drummond DA, Raval A, Wilke CO (2005) A single determinanat dominates the rate of yeast protein evolution. Mol Biol Evol 23: 327–337. 16237209 10.1093/molbev/msj038

[85] Subramanian S, Kumar S (2004) Gene expression intensity shapes evolutionary rates of the proteins encoded by the vertebrate genome. Genetics 168: 373–381. 15454550 10.1534/genetics.104.028944 PMC1448110

[86] Pal C, Papp B, Hurst LD (2001) Highly expressed genes in yeast evolve slowly. Genetics 158: 927–931. 11430355 10.1093/genetics/158.2.927 PMC1461684

[87] Duret L, Mouchiroud D (2000) Determinants of substitution rates in mammalian genes: expression pattern affects selection intensity but not mutation rate. Mol Biol Evol 17: 68–74. 10666707 10.1093/oxfordjournals.molbev.a026239

[88] Rocha EPC, Danchin A (2004) An analysis of determinants of amina acid substitution rates in bacterial proteins. Mol Biol Evol 21: 108–116. 14595100 10.1093/molbev/msh004

[89] Liao BY, Zhang JZ (2006) Low rates of expression profile divergence in highly expressed genes and tissue-specific genes during mammalian evolution. Mol Biol Evol 23: 1119–1128. 16520335 10.1093/molbev/msj119

[90] Mank JE, Hultin-Rosenberg L, Axelsson E, Ellegren H (2007) Rapid evolution of female-biased, but not male-biased, genes expressed in the avian brain. Mol Biol Evol 24: 2698–2706. 17893399 10.1093/molbev/msm208

[91] Connallon T, Clark AG (2011) The resolution of sexual antagonism by gene duplication. Genetics 187: 919–937. 21220356 10.1534/genetics.110.123729 PMC3063682

[92] Vicoso B, Charlesworth B (2009) The deficit of male-biased genes on the *D. melanogaster* X chromosome is expression-dependent: a consequence of dosage compensation? J Mol Evol 68: 576–583. 19407921 10.1007/s00239-009-9235-4

[93] Seoighe C, Wolfe KH (1999) Yeast genome evolution in the post-genome era. Curr Opin Microbiol 2: 548–554. 10508730 10.1016/s1369-5274(99)00015-6

[94] Emerson JJ, Cardoso-Moreira M, Borevitz JO, Long M (2008) Natural selection shapes genome-wide patterns of copy-number polymorphism in *Drosophila melanogaster* . Science 320: 1629–1631. 18535209 10.1126/science.1158078

[95] Mank JE, Ellegren H (2009) Sex-linkage of sexually antagonistic genes is predicted by female, but not male, effects in birds. Evolution 63: 1464–1472. 19154378 10.1111/j.1558-5646.2009.00618.x

[96] Arunkumar KP, Mita K, Nagaraju J (2009) The silkworm Z chromosome is enriched in testis-specific genes. Genetics 182: 493–501. 19332883 10.1534/genetics.108.099994 PMC2691758

